# The integral role of the clinical pharmacist in drug-assisted intubation at a newly established children’s major trauma center

**DOI:** 10.1007/s11096-021-01262-x

**Published:** 2021-04-13

**Authors:** Kevin Enright, Shazia Akram, Amna Hussain, Colin V. E. Powell

**Affiliations:** 1 Department of Emergency Medicine, Sidra Medical and Research Center, Doha, Qatar; 2 Division of Population Medicine, School of Medicine, Cardiff University, Cardiff, UK

**Keywords:** Guideline, Pediatric emergency medicine, Pharmacists, Rapid sequence induction and intubation, Trauma centers

## Abstract

This commentary outlines how the clinical pharmacist can support the safe administration of emergency medications in trauma anesthesia for seriously injured children. Promoting the professional development of the clinical pharmacist provided an opportunity to strengthen a key step in our trauma care pathway. We describe the implementation of this process in a new hospital, which was to become the designated children’s trauma center for an entire country. Although the literature documents the use of pharmacists in emergency intubation, ours was a unique set of circumstances, where empowering the pharmacist in frontline clinical care provided additional quality assurance for rapid sequence induction and intubation in trauma. Medical simulation was a core part of socializing the advanced clinical practice role of pharmacy within the trauma team. It was our experience that the pharmacist helps to promote confidence and decision making among other members of the trauma team.

## Impacts on practice

- Continuing professional development includes opportunities for pharmacists to integrate into frontline critical care teams, including pediatric trauma teams.
- The clinical pharmacist plays a key role in facilitating safe and consistent medication management during rapid sequence induction and intubation in pediatric major trauma.
- Pharmacists at trauma centers can be responsible for establishing drug protocols, providing clinical decision support and auditing compliance. Best practice standards emerge from meeting the challenges of working in new clinical settings.
- Medical simulation can form the basis for establishing the pharmacist’s advanced practice role within critical care teams.

## Introduction

In June 2018, Sidra Medicine in Doha, Qatar, a 400-bed tertiary women’s and children’s hospital, and the largest of its kind in the Middle East, launched its Pediatric Emergency Medicine (PEM) program. This newly established pediatric emergency department (PED) would receive all cases of pediatric major trauma for the entire country. Qatar has a population of 2.8 million, 500,000 of whom are children. The new trauma center expected to receive approximately 300 cases of major trauma per annum, including up to twenty seriously injured children requiring emergency intubation.

Drug-assisted intubation in major trauma is a high-risk, time-critical intervention, often undertaken in unstable patients [1, 2]. The traditional role of clinical pharmacy included procurement, preparation and provision of medications [3]. The contemporary role now includes clinical practice in emergency medicine. This is a shift in intensity of clinical experience for the pharmacist. This expanded role has been supported by the Institute of Medicine [4]. In our center, the newly appointed PED medical and nursing staff had previously trained and worked in a variety of different healthcare systems worldwide. During preliminary discussions, it was recognized that there was the potential for a wide range of non-standardized approaches to the delivery of trauma room anesthesia [4, 5]. We believed that as part of the standard of care for this department, consistent practice in delivery of rapid sequence induction and intubation would lead to safer outcomes for patients [6]. In this setting, the clinical pharmacist would prepare medications alongside the trauma room nurses [7]. They would be core members of the hospital trauma team and would be a real-time resource for the entire clinical team [8].

The aims of this paper are to document the role of the clinical pharmacist in supporting drug-assisted intubation in pediatric major trauma, to describe the process by which this was implemented, and to outline the benefits of having a clinical pharmacist as an integral part of a newly established trauma system.

## Pre activation preparation

**Fig. 1:**
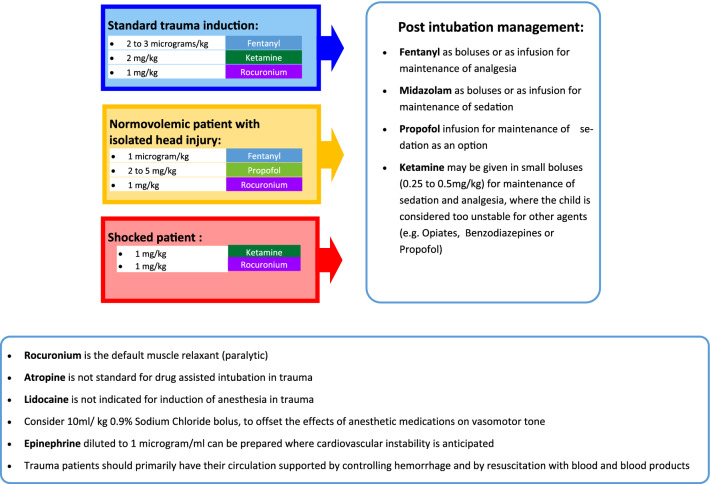
Guideline for delivery of anesthetic medications in rapid sequence induction and intubation in trauma

Working alongside emergency physicians and anesthesiologists, the clinical pharmacist developed a guideline for delivery of anesthetic medications in rapid sequence induction and intubation in trauma (Fig. 1) [9]. It was agreed that the default medication regimen for intubation would be two to three micrograms per kilogram of fentanyl, two milligrams per kilogram of ketamine and one milligram per kilogram of rocuronium, intravenously [10, 11]. For patients suspected of hypovolemic shock, there was the option to reduce the doses of fentanyl or ketamine or both, in a pre-determined ratio [12]. A further option was to undertake intubation using intravenous propofol as the induction agent in stable patients with isolated head injuries [13]. Ours is a more comprehensive protocol than has been published previously [9]. Ten core principals of pharmacology for intubation in trauma were agreed and implemented as a clinical practice guideline (Fig. 1). The medication doses were rounded to the nearest five micrograms (or five milligrams) to facilitate quicker, safer preparation and administration, and more reliable therapeutic efficacy. The guideline was consistent with standard practice at other trauma centers and pre-hospital care systems, including helicopter emergency medicine services (HEMS), which have been shown to be safe [14].

The clinical pharmacist developed weight and age based, color-coded medication flowcharts and prepared resource folders. Additional resources included a pre-printed prescription template for use in each trauma activation, listing the standard medications as a prompt for each trauma case. Therefore, the drugs could be drawn-up and checked in advance of the patient’s arrival.

Medical simulation formed a core component of the hospital’s preparation for launching the national children’s major trauma center over a six month period. Simulation is well established in clinical training programs [15]. As a member of the responding trauma team, the pharmacist participated in trauma simulations.

## Opening the PED to major trauma

Over 100 trauma call activations were simulated using manikins, before the hospital opened its doors to major trauma. A clinical pharmacist attended each simulation and participated as a core member of the trauma team. Their duties in trauma simulation, and subsequently in real life, included recommending, preparing and verifying medications, and prompting the other trauma team members to follow the agreed guideline.

**Fig. 2:**
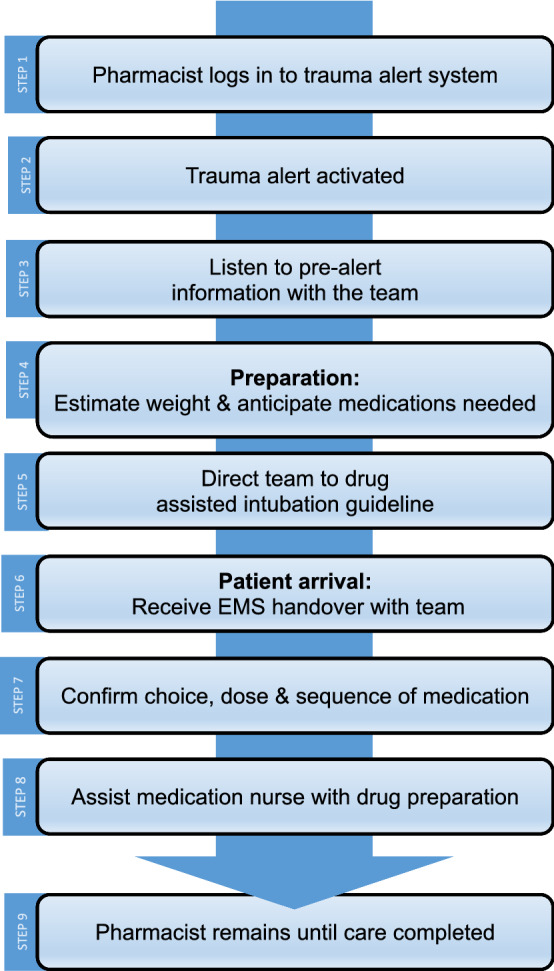
Flowchart of pharmacist workflow when responding to trauma alerts

Following activation of major trauma, the clinical pharmacist provided ongoing departmental pharmacology education to trauma team members during daily clinical shifts. They would also teach and support staff during trauma calls, reiterating the rationale for use of particular anesthetic medications and the relevant weight based dosing, including using ideal body weight estimations. The child’s weight was estimated prior to arrival, and confirmed once they were present in the trauma room (Fig. 2).

Two dedicated clinical pharmacists were allocated to be present in ED for 70 h per week and 15 additional pharmacists also responded as trauma team members. This allowed for 24-h provision of clinical pharmacy support. The two lead pharmacists provide mentoring and educational support to the wider pharmacy team and the nurses on the trauma team rely on their presence. Over time, this has helped to build the confidence of the nursing team with regard to drug-assisted intubation. A key responsibility of the clinical pharmacist is participation in the clinical governance functions of the hospital’s trauma program. Their duties include participation in trauma call debriefs, weekly review of trauma call documentation, and maintaining a database of all drug-assisted intubations to detect any notable deviations from the guideline.

## Description of clinical activity

As the population has increased in Qatar, the number of severely injured children has risen [16] and there were 500 trauma calls in the first 12 months following activation from May 2019. Our center received cases from 0 to 14 years. 24 children were severely injured with an injury severity score (ISS) greater than 15 [17], which was classified as tier 1 trauma. Of these, 19 were male. Children presented with the following mechanisms of injury: motor vehicle accident (MVA), hanging, falls, drowning, burns and penetrating injury. 18 children died. The female fatalities have all been due to asphyxiation from drownings or hangings. The male fatalities include blunt trauma mechanisms (falls and MVAs). 15 children were admitted to the pediatric intensive care unit (PICU), two children underwent interventional radiology for life threatening hemorrhage and ten of the children went directly from the trauma room to the operating room.

There were 34 children (27 male) who underwent intubation by the trauma team. 14 children were intubated without drugs: these were children in traumatic cardiac arrest. There were two cases who underwent drug-assisted intubation where their management deviated from the guideline (Fig. 1). One patient was a child with facial burns who received suxamethonium and propofol. This provided no analgesia on induction and the suxamethonium wore off prior to effective post intubation sedation. Following this incident, teaching was provided to nursing staff and ED physicians on our guideline with focus on pre-intubation and post intubation management. The team has been reminded to not use suxamethonium due to its short duration of action and to bolus midazolam as post intubation sedation management, if the patient is at risk of waking up too soon.

The other child had multiple injuries and received propofol and rocuronium for intubation. This led to an episode of hypotension, which may have been avoided using the standard fentanyl, ketamine and rocuronium regimen. In order to manage propofol-induced hypotension more quickly, we chose epinephrine 1 µg/mL as our default sympathomimetic agent and supplied guidance on how to prepare this with minimal dilutions and calculations.

## Discussion

Clinical pharmacists have been present in trauma teams before, particularly in North America and several publications document the expanding role of pharmacists in trauma teams [4, 18]. This is the first detailed description of their enhanced role in the safe administration of anesthesia for rapid sequence induction and intubation for pediatric major trauma in a newly emerging system. In our system, the input of the pharmacist facilitated the use of a greater range of intubation medications, which is yet another extension of clinical pharmacy practice [8]. In this commentary, we have shared our experiences of putting the clinical pharmacist at the center of the process.

The activation of the new pediatric trauma program in Qatar followed months of intensive preparation including simulation training across all services in the hospital. Prior to receiving the first major trauma patient, the clinical pharmacist and the lead for major trauma identified the need to standardize the choice and dosage of medications in drug- assisted intubation. This was particularly important when recognizing the variability in practice from our globally recruited cohort of healthcare staff, but could equally be applied to other healthcare systems, with the aim of reducing adverse events and supporting clinical decision making [19].

The PED clinical pharmacist was a key link between the trauma team members and the new clinical guidelines, drawing on expertise from healthcare systems around the world. This report has outlined the integral role the clinical pharmacists have played in establishing and monitoring the clinical pathway, especially in being prescriptive about drug regimens. This has allowed the professional profile of the pharmacist to advance from drawing up and checking medications, to co-leading in decision making and influencing the choice of medications in a high acuity, critical situation. A consequence of the implementation of the guideline for drug-assisted intubation in trauma, was that it became the default for the critically unwell medical cases also. The resuscitation teams during weekly meetings, described feeling confident using the trauma guideline in comparable situations. Further to this, using a pre-printed prescription template as a cognitive aid resulted in a high compliance rate with the guideline for rapid sequence induction and intubation.

A standardized guideline has helped to deliver drug-assisted intubation in trauma resuscitation during the additional challenges posed by the COVID-19 pandemic. Our PED pharmacists were not routinely provided with N95 masks and so remained outside the trauma room, to assist remotely. Our teams’ familiarity with the guideline ensured efficient and practiced management of drug-assisted intubation.

The volume of trauma is likely to increase as the population grows, underpinning the benefit of a standardized guideline to maintain consistency of approach [9, 15].

## Conclusion

The clinical pharmacist played an integral role in the safe and consistent delivery of anesthetic medications during drug-assisted intubation at our newly established pediatric trauma center. This integral role has been described and documented previously citing examples from Europe, Australia and the Middle East [3, 4]. The pharmacist contributed to the education, training, and governance functions of the trauma program. In addition, their presence as a member of the trauma team promoted optimization of pharmacotherapy.
